# Mendelian randomization analysis of the causal association of bone mineral density and fracture with multiple sclerosis

**DOI:** 10.3389/fneur.2022.993150

**Published:** 2022-09-15

**Authors:** Yu Yao, Feng Gao, Yanni Wu, Xin Zhang, Jun Xu, Haiyang Du, Xintao Wang

**Affiliations:** Department of Orthopedics, The Second Affiliated Hospital of Harbin Medical University, Harbin, China

**Keywords:** multiple sclerosis, bone mineral density, fracture, genome-wide association study, Mendelian randomization

## Abstract

Multiple sclerosis (MS) is a neurodegenerative disorder and an autoimmune disease. Until now, observational studies have indicated the association of bone mineral density (BMD) and fracture with the risk of MS. However, these studies indicated inconsistent findings. Until now, genome-wide association studies (GWAS) have been conducted in BMD, fracture, and MS, which provide large-scale datasets to investigate the causal association of BMD and fracture with the risk of MS using the Mendelian randomization (MR) study. Here, we performed an MR study to clarify the causal association between BMD/fracture and the risk of MS using large-scale publicly available GWAS datasets from BMD, fracture, and MS. We first evaluated the bidirectional causal effects of BMD and MS. The main analysis method inverse-variance weighted (IVW) showed no significant causal effect of BMD on the risk of MS (β = 0.058, and *p* = 1.98E-01), and MS on the risk of BMD (β = −0.001, and *p* = 7.83E-01). We then evaluated the bidirectional causal effects of fracture and MS. However, we only identified a significant causal effect of fracture on the risk of MS using IVW (β = −0.375, *p* = 0.002), but no significant causal effect of MS on the risk of the fracture using IVW (β = 0.011, *p* = 2.39E-01). Therefore, our main analysis method IVW only found a significant causal effect of fracture on MS using the threshold for the statistically significant association *p* < 0.05/4 = 0.0125. Meanwhile, multivariable MR analyses showed that the causal effect of fracture on MS was independent of smoking, drinking, and obesity, but dependent on BMD. In summary, our MR analysis demonstrates that genetically increased fracture may reduce the risk of MS. Our findings should be further verified and the underlying mechanisms should be further evaluated by future studies.

## Introduction

Multiple sclerosis (MS) is considered to be a neurodegenerative disorder and an autoimmune disease (1). Until now, observational studies have indicated the association of bone mineral density (BMD) and fracture with the risk of MS. However, these studies indicated inconsistent findings. Simonsen et al. selected 91 patients with MS with the disease for at least 10 years, and analyzed their BMD data from the spine, hip, and total body, as well as the biochemical measures of bone metabolism (2). Their results indicated that 74.7% of the 91 patients with MS had the relative low BMD (osteopenia or osteoporosis) (2). Bisson at al. analyzed the BMD screening data from 783 patients with MS and 3,915 age, sex, region of residence, and date of BMD screening matched controls (3). They identified lower mean BMD in MS cases at three sites, including femoral neck, total hip, and lumbar spine, compared with the controls (3). Meanwhile, the MS cases had the higher prevalence of osteoporosis compared with controls (3). Olsson et al. used a novel analytical tool called trabecular bone score to measure the bone microarchitecture in 260 patients with MS (4). They found that there was no significant difference in trabecular bone scores between the MS cases and age-matched reference population (4).

In addition to BMD, multiple studies have evaluated the association between fracture and MS. Bazelier et al. conducted a population-based cohort study to evaluate the risk of fractures in 2,415 patients with MS and 6 year of birth, sex, and practice matched controls (5). They found that patients with MS had significantly increased risk of fracture (5). A record-linkage study indicated significantly increased risk of fracture in patients with MS (6). Bazelier et al. analyzed the data from 5,565 patients with MS and 33,360 controls in the UK General Practice Research Database (7). They identified patients with MS as having a 2.78-fold increased risk of hip fracture, compared with controls (7). A large-scale meta-analysis in nearly 9,000,000 subjects further supported the significant association between MS and increased risk of fracture (8).

Multiple sclerosis, BMD, and fracture are three complex human diseases or phenotypes, which are caused by genetic and environmental factors and their interactions (9–11). Until now, genome-wide association studies (GWAS) have been conducted in BMD and fracture (9–11). Using the genetic variants as the instrumental variables, Mendelian randomization (MR) studies have been widely conducted to evaluate the causal association of BMD and fracture with kinds of human diseases and phenotypes (12–17). However, it is currently unknown about the causal association of BMD and fracture with the risk of MS. Interestingly, the publicly available MS GWAS provide large-scale datasets to investigate the causal association of BMD and fracture with the risk of MS (11).

## Methods

### Study design

Our study is based on a bidirectional MR design, which has been well established and was applied to different kinds of MR studies (18–21). Here, we performed a MR study to clarify the bidirectional causal association between BMD/fracture and the risk of MS using large-scale publicly available GWAS datasets, as provided in Table 1. MR has three assumptions: (1) instrumental variables (genetic variants) are strongly associated with an exposure, generally the genome-wide significance (*p* <5.00E-08); (2) instrumental variables are independent of confounders; and (3) instrumental variables affect one outcome only *via* the exposure path (24).

**Table 1:** Genome-wide association studies (GWAS) datasets selected in the current Mendelian Randomization (MR) study.

**GWAS datasets**	**Consortium**	**Case number**	**Control number**	**References**
MS	International Multiple Sclerosis Genetics Consortium (IMSGC)	14,802	26,703	(25)
BMD	GEnetic Factors for OSteoporosis consortium (GEFOS)	NA	142,487	(10)
Fracture	GEnetic Factors for OSteoporosis consortium (GEFOS)	37,857	227,116	(11)
Smoking (cigarettes per day)	GWAS and Sequencing Consortium of Alcohol and Nicotine use consortium (GSCAN)	337,334	NA	(22)
Drinking (drinks per week)	GWAS and Sequencing Consortium of Alcohol and Nicotine use consortium (GSCAN)	941,280	NA	(22)
Obesity (BMI)	Genetic Investigation of ANthropometric Traits (GIANT) consortium	241,258	NA	(23)

### MS genetic variants and MS GWAS dataset

We selected 200 independent MS autosomal non-major histocompatibility complex (MHC) genetic variants with the genome-wide significance (*p* <5.00E-08) as the potential instrumental variables, which were newly identified by the International Multiple Sclerosis Genetics Consortium (IMSGC) (25). IMSGC is a large-scale meta-analysis of MS GWAS datasets from three stages, including the discovery stage (14,802 MS cases and 26,703 controls), the MS Chip stage (20,360 MS subjects and 19,047 controls), and the ImmunoChip stage (12,267 MS subjects and 22,625 control), which consisted of 47,429 MS cases and 68,374 control individuals (2019). The summary results about the 200 autosomal non-MHC genetic variants are provided in Supplementary Table 1. Only the full MS GWAS summary results from the IMSGC discovery stage are publicly available, which is a meta-analysis of 15 MS GWAS datasets, including 14,802 MS cases and 26,703 controls. The original study provides more detailed information (2019).

### BMD genetic variants and BMD GWAS dataset

We selected 307 conditionally independent (*r*^2^ < 0.1) heel BMD genetic variants reaching genome-wide significance, which were identified by a large-scale BMD GWAS in 142,487 individuals from the UK Biobank (10). It is estimated that these 307 genetic variants can explain about 12% of the heel BMD variance (10). The summary results about the 307 heel BMD genetic variants are provided in Supplementary Table 2. Meanwhile, the full heel BMD GWAS summary results from these 142,487 individuals are publicly available.

### Fracture genetic variants and fracture GWAS dataset

We selected 15 independent (*r*^2^ < 0.05) fracture genetic variants with genome-wide significance (*p* <5.00E-08) as the potential instrumental variables, which were newly identified by the GEnetic Factors for OSteoporosis consortium (GEFOS) (11). The fracture GWAS included a total of 185,057 cases and 377,201 controls from a large-scale meta-analysis of the discovery stage (37,857 cases and 227,116 controls) and the replication stage (147,200 cases and 150,085 controls) (11). Table 2 provides the detailed information about these 15 fracture genetic variants. Only the full fracture GWAS summary results from the GEFOS discovery stage are publicly available, which is a meta-analysis of 25 fracture GWAS datasets including 37,857 cases and 227,116 controls from Europe (*n* = 15), North America (*n* = 8), Australia (*n* = 1), and East Asia (*n* = 1), as provided in the original study (11).

**Table 2:** Detailed information about 15 fracture genetic variants.

**SNP**	**Locus**	**Candidate gene**	**EA**	**EAF**	**Odds ratio (95% CI)**	***P***
rs4233949	2p16.2	*SPTBN1*	G	0.61	1.03 (1.02–1.04)	2.8 × 10^−14^
rs430727	3p22.1	*CTNNB1*	T	0.45	1.03 (1.02–1.04)	5.0 × 10^−12^
rs10457487	6q22.33	*RSPO3*	C	0.51	1.05 (1.04–1.06)	4.8 × 10^−28^
rs2982570	6q25.1	*ESR1*	C	0.58	1.04 (1.03–1.05)	4.5 × 10^−19^
rs2908007	7q31.31	*WNT16, CPED1*	A	0.6	1.06 (1.05–1.07)	2.3 × 10^−39^
rs6465508	7q21.3	*C7orf76, SHFM1*	G	0.34	1.04 (1.03–1.05)	2.0 × 10^−19^
rs6959212	7p14.1	*STARD3NL*	T	0.34	1.03 (1.02–1.04)	8.8 × 10^−10^
rs1548607	7p12.1	*GRB10, COBL*	G	0.32	1.03 (1.02–1.05)	4.7 × 10^−10^
rs7851693	9q34.11	*FUBP3*	G	0.35	1.04 (1.03–1.05)	5.0 × 10^−19^
rs11003047	10q21.1	*MBL2/DKK1*	G	0.11	1.09 (1.07–1.10)	9.5 × 10^−33^
rs3736228	11q13.2	*LRP5*	T	0.15	1.06 (1.05–1.08)	1.0 × 10^−21^
rs1286083	14q32.12	*RPS6KA5*	T	0.82	1.05 (1.04–1.06)	1.6 × 10^−17^
rs2741856	17q21.31	*SOST, DUSP3, MEOX1*	G	0.92	1.10 (1.07–1.11)	3.1 × 10^−25^
rs4635400	18p11.21	*FAM210A, RNMT*	A	0.36	1.04 (1.03–1.05)	1.1 × 10^−18^
rs9980072	21q22.2	*ETS2*	G	0.73	1.04 (1.03–1.05)	3.4 × 10^−13^

EA, effect allele; EAF, effect allele frequency.

### MR analysis and pleiotropy analysis

We selected the large-scale BMD, fracture, and MS GWAS summary datasets, as well as their corresponding genetic variants reaching genome-wide significance (*p* <5.00E-08) as the potential instrumental variables to perform the MR analysis. We conducted the MR analysis using an inverse-variance weighted (IVW) (26), a weighted median (26), an MR-Egger test (27), a contamination mixture method (28), and a Mendelian Randomization Pleiotropy RESidual Sum and Outlier (MR-PRESSO) test (29). The IVW method is selected as the main method, while the weighted median, MR-Egger, contamination mixture method, and MR-PRESSO are selected as the supplementary methods (19, 20, 29–32). These MR methods have widely been described in recent MR studies (19, 20, 30–32).

The IVW method is the most efficient MR method if all genetic variants are valid instruments; in other words, there is no evidence of pleiotropy (33). However, IVW will be biased if some genetic variants are invalid instruments; in other words, evidence of pleiotropy (33). We further selected other methods to detect or adjust for pleiotropy, including the weighted median, MR-Egger, contamination mixture method, and MR-PRESSO as the supplementary methods (19, 20, 30–32). The MR-Egger method allows some or even all genetic variants to be invalid instruments but requires these genetic variants to satisfy the instrument strength independent of direct effect (InSIDE) assumption (33). MR-Egger could detect the pleiotropy using the MR-Egger intercept test and correct the presence of pleiotropy (27). The weighted median could provide a consistent estimate of the causal effect when more than 50% of the weight is contributed by instrumental variables (26). MR-PRESSO has several advantages over MR-Egger (29). MR-PRESSO not only could evaluated horizontal pleiotropy but also identify and remove pleiotropic genetic variants (29). In addition to the MR-Egger intercept test and MR-PRESSO, we also selected the Cochran's *Q* statistic together with the *I*^2^ statistic to evaluate heterogeneity due to pleiotropy (34). He and colleagues have provided more detailed information about Cochran's *Q* statistic and *I*^2^ statistic (35).

For the statistically significant association, we further performed a multivariable MR analysis to adjust for major confounders, such as smoking, drinking, and obesity, as provided in Table 1. Meanwhile, fracture acts as a main detrimental consequence of low BMD. Therefore, we also performed a multivariable MR analysis using the multivariable IVW method to adjust for the effect of BMD when evaluating the causal effects of fracture on MS. All statistical tests were calculated using R Package “MendelianRandomization” (version 0.5.1) (36) and R 4.05. The thresholds for the statistically significant association and suggestive association are *p* < 0.05/4 = 0.0125 and *P* < 0.05, respectively.

### Power analysis

Power analysis was performed using mRnd (Power calculations for Mendelian Randomization) (37). Meanwhile, mRnd requires the proportion of variance in the exposure explained by the genetic variants *R*^2^, which was calculated using


$$\begin{array}{l} R^{2}\;=\;\underset{j\;=\;1}{\sum }k2\;*\;MAF\;*\;\left(1-MAF\right)\;*\;\beta_{j}2 \end{array}$$

Where, β_*j*_, the effect size for genetic variant; MAF, the minor allele frequency for genetic variant; and *K*, the number of genetic variants (38). Liu and colleagues have provided a more detailed description of the power analysis method (30).

## Results

### Bidirectional effects of BMD and MS

**Figure 1:**
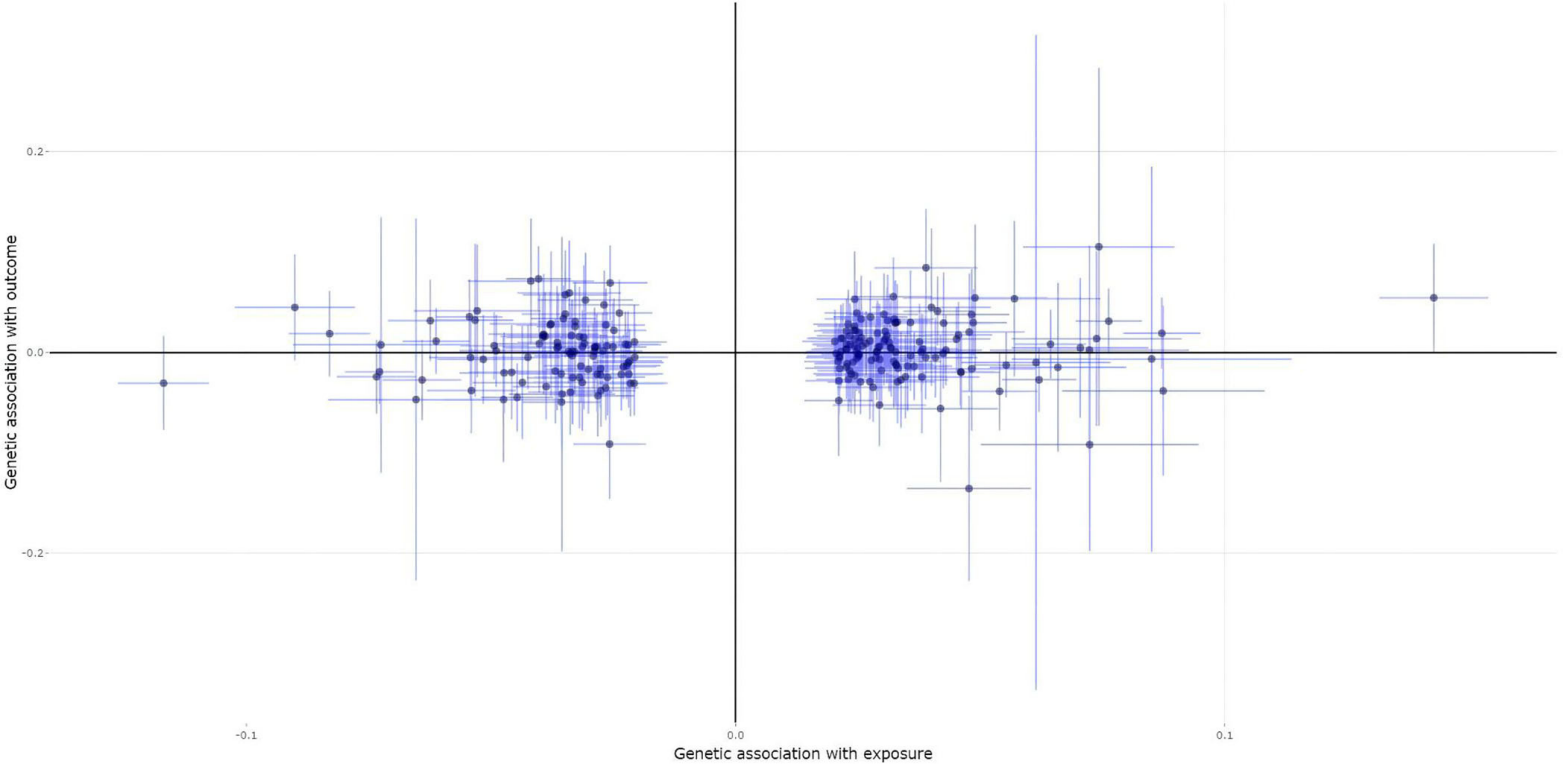
The scatter plot for the Mendelian randomization (MR) analysis showing the causal estimates of bone mineral density (BMD) on the risk of multiple sclerosis (MS) using the contamination mixture method.

A total of 222 of the 307 heel BMD genetic variants were available and their corresponding summary statistics were further extracted from the MS GWAS summary dataset. An MR analysis using IVW and MR-Egger showed no significant causal effect of BMD on the risk of MS. Interestingly, the weighted median and contamination mixture methods suggest a significant causal effect of BMD on the risk of MS with beta = 0.122, 95% *CI*: 0.002–0.243, *p* = 0.047; and β = 0.17, 95% *CI*: 0.07–0.29, *p* = 0.00235, respectively. Figure 1 is the scatter plot for the MR analysis showing the causal estimates of BMD on the risk of MS using the contamination mixture method.

An MR-Egger intercept test identified no evidence of pleiotropy (intercept = 0, and *p* = 0.934), as provided in Table 4. However, the heterogeneity test (*I*^2^ = 36.2% and *p* = 0.0000) and the MR-PRESSO Global Test (*p* = <5e-04) show evidence of pleiotropy (Table 4). The MR-PRESSO test and the MR-PRESSO Outlier-corrected test still show no significant causal effect of BMD on the risk of MS (Table 3).

**Table 3:** An MR analysis results about the causal association of bone mineral density (BMD) and fracture with multiple sclerosis (MS).

**Effects**	**Methods**	**Beta**	**95% CI (lower)**	**95% CI (upper)**	***P-*value**
BMD on MS	Weighted median	0.122	0.002	0.243	4.70E-02
	IVW	0.058	−0.03	0.147	1.98E-01
	MR-Egger	0.067	−0.154	0.287	5.54E-01
	Contamination mixture method	0.17	0.07	0.29	**2.35E-03**
	MR-PRESSO	0.058	−0.030	0.147	2.00E-01
	MR-PRESSO Outlier-corrected	0.079	−0.006	0.164	6.87E-02
MS on BMD	Weighted median	0.01	0.001	0.02	2.90E-02
	IVW	−0.001	−0.011	0.008	7.83E-01
	MR-Egger	0.005	−0.028	0.038	7.69E-01
	Contamination mixture method	0.01	0.01	0.01	1.96E-02
	MR-PRESSO	−0.001	−0.011	0.008	7.84E-01
	MR-PRESSO Outlier-corrected	0.005	−0.003	0.012	2.33E-01
Fracture on MS	Weighted median	−0.371	−0.671	−0.072	1.50E-02
	IVW	−0.375	−0.617	−0.134	**2.00E-03**
	MR-Egger	−0.157	−0.936	0.622	6.93E-01
	Contamination mixture method	−0.53	−0.95	−0.03	3.86E-02
	MR-PRESSO	−0.375	−0.617	−0.134	**8.69E-03**
	MR-PRESSO Outlier-corrected	NA	NA	NA	NA
MS on fracture	Weighted median	0.011	−0.015	0.036	4.19E-01
	IVW	0.011	−0.007	0.029	2.39E-01
	MR-Egger	−0.004	−0.062	0.053	8.88E-01
	Contamination mixture method	0	−0.03	0.01	1.00E+00
	MR-PRESSO	0.011	−0.007	0.029	2.41E-01
	MR-PRESSO Outlier-corrected	NA	NA	NA	NA

IVW, inverse-variance weighted; the thresholds for the statistically significant association and suggestive association are *p* < 0.05/4 = 0.0125 and *p* < 0.05, respectively. The bold values mean the statistically significant association.

**Figure 2:**
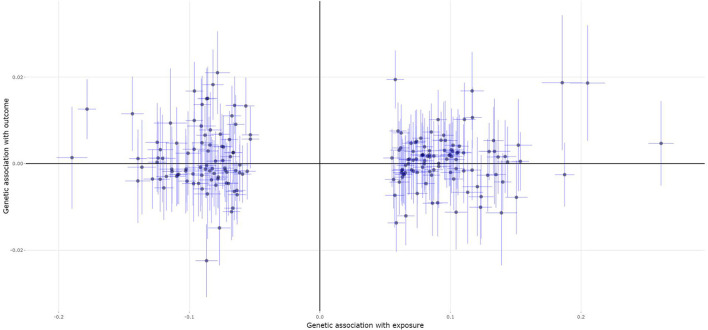
The scatter plot for the MR analysis showing the causal estimates of MS on BMD using the contamination mixture method.

A total of 189 of the 200 MS genetic variants were available and their corresponding summary statistics were further extracted from the BMD GWAS summary dataset. An MR analysis using IVW and MR-Egger also showed no significant causal effect of MS on the risk of BMD. Interestingly, both the weighted median and contamination mixture methods showed suggestive causal effect of MS on the risk of BMD with β = 0.01, 95% *CI*: 0.001–0.02, *p* = 0.029; and β = 0.01, *p* = 0.0196, respectively. Figure 2 is the scatter plot for the MR analysis showing the causal estimates of MS on BMD using the contamination mixture method.

An MR-Egger intercept test showed no evidence of pleiotropy (intercept = −0.001, and *p* = 0.694), as provided in Table 4. However, the heterogeneity test (*I*^2^ = 64.6% and *p* = 0.0000) and the MR-PRESSO Global test (*p* = <5e-04) show evidence of pleiotropy (Table 4). The MR-PRESSO test and the MR-PRESSO Outlier-corrected test still show no significant causal effect of BMD on the risk of MS (Table 3).

**Table 4:** Pleiotropy analysis about the causal association of BMD and fracture with MS.

**Effects**	**Heterogeneity test**		**MR-Egger intercept test**		**MR-PRESSO**
	***I* ^2^**	***P*-value**	**Intercept**	***P*-value**	**Global test *P*-value**
BMD on MS	36.2%	0.0000	0	0.934	<5e-04
MS on BMD	64.6%	0.0000	−0.001	0.694	<5e-04
**Fracture on MS**	**22.9%**	**0.1992**	–**0.01**	**0.562**	**NA**
MS on Fracture	18.9%	0.0325	0.001	0.589	0.0355

The thresholds for the statistically significant pleiotropy is *p* < 0.05.

### Bidirectional effects of fracture and MS

**Figure 3:**
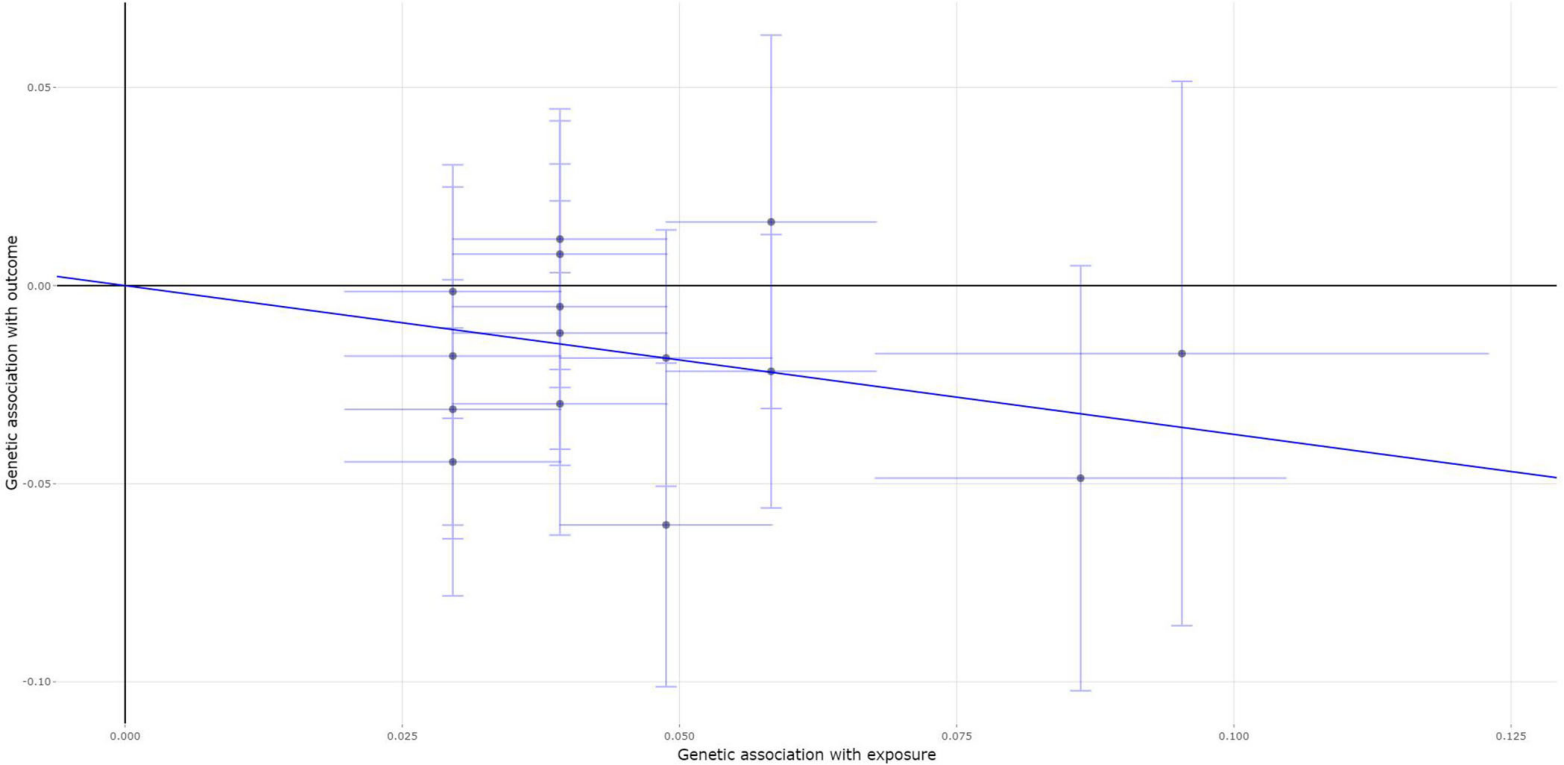
The scatter plot for the MR analysis showing the causal estimates of fracture on the risk of MS using the inverse-variance weighted (IVW) method.

All the selected 15 fracture genetic variants were available and their corresponding summary statistics were further extracted from the MS GWAS summary dataset. An MR analysis using IVW (β = −0.375, 95% *CI*: [−0.617, −0.134], *p* = 0.002), the weighted median (β = −0.371, 95% *CI*: [−0.671, −0.072], *p* = 0.015), and the contamination mixture method (β = −0.53, 95% *CI*: [-0.95, −0.03], *p* = 0.0386) indicated a statistically significant or suggestive causal effect of fracture on the risk of MS. Only MR-Egger showed no significant causal effect of fracture on the risk of MS, as provided in Table 2. Figure 3 is the scatter plot for the MR analysis showing the causal estimates of fracture on the risk of MS using IVW.

An MR-Egger intercept test showed no evidence of pleiotropy (intercept = −0.01, and *p* = 0.562), as provided in Table 4. Meanwhile, the heterogeneity test (*I*^2^ = 22.9% and *P* = 0.1992) and the MR-PRESSO Global test show that there is no evidence of pleiotropy (Table 4). The MR-PRESSO test still show significant causal effect of BMD on the risk of MS (Table 3).

**Figure 4:**
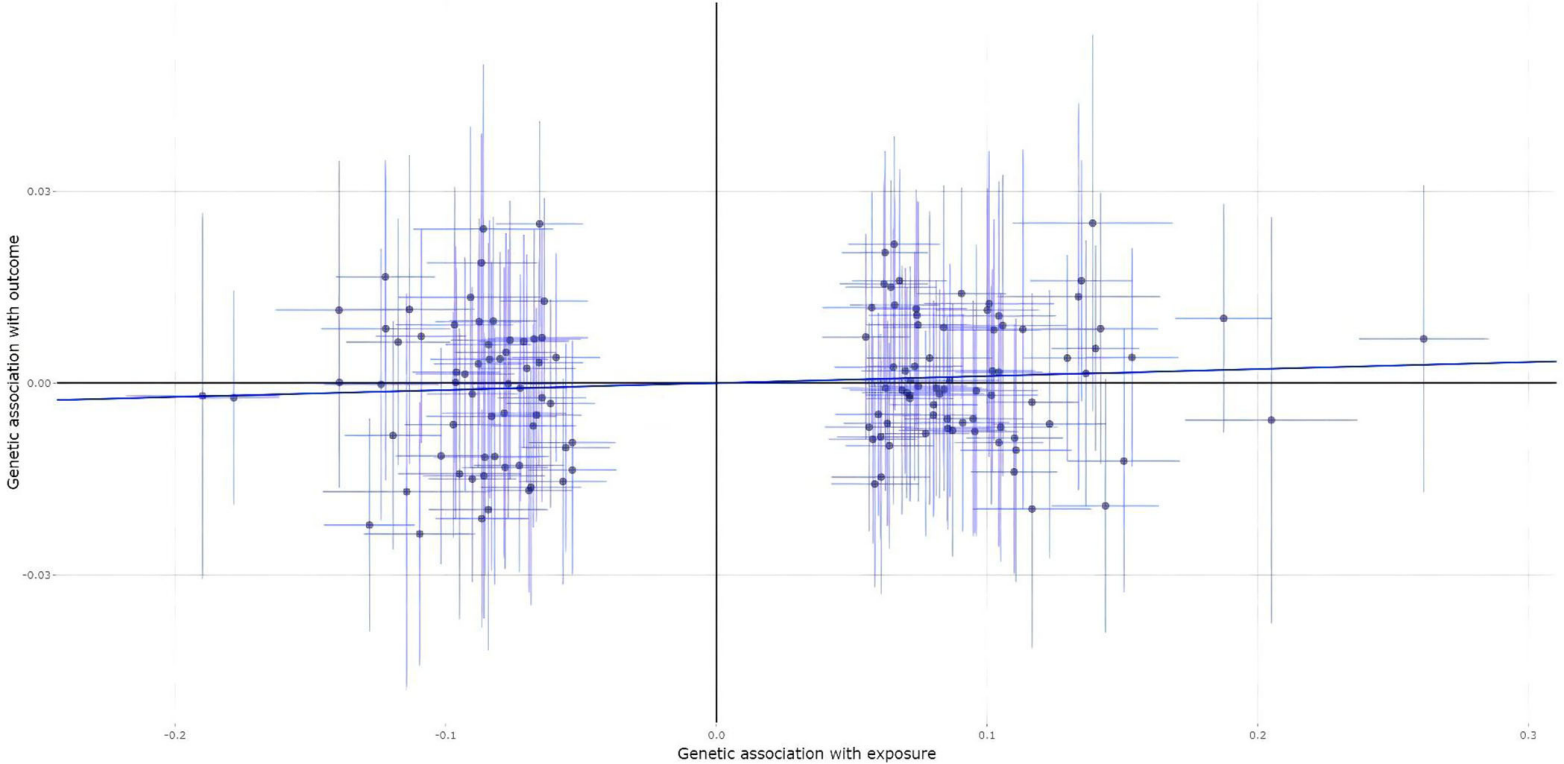
The scatter plot for the MR analysis showing the causal estimates of MS on fracture using the IVW method.

A total of 139 of the 200 MS genetic variants were available and their corresponding summary statistics were further extracted from the fracture GWAS summary dataset. However, all these four MR analysis methods, such as the IVW, weighted median, MR-Egger, and contamination mixture methods indicated no significant causal effect of MS on the risk of fracture, as provided in Table 2. Figure 4 is the scatter plot for the MR analysis showing the causal estimates of MS on fracture using the IVW method.

The MR-Egger intercept test showed no evidence of pleiotropy (intercept = 0.001, and *p* = 0.589), as provided in Table 4. Meanwhile, the heterogeneity test (*I*^2^ = 18.9% and *p* = 0.0325) and the MR-PRESS Global test (*p* = 0.0355) show evidence of pleiotropy (Table 4). The MR-PRESSO test and the MR-PRESSO Outlier-corrected test still show no significant causal effect of MS on the risk of fracture (Table 3).

### Power analysis

Using the threshold for the statistically significant association *p* < 0.05/4 = 0.0125, our main analysis method IVW only found a significant causal effect of fracture on MS. Therefore, our power analysis only focused on this association. These 15 fracture genetic variants explain 1.23% of variance in the fracture (*R*^2^ = 1.23%). Power analysis using the mRnd showed that our MR study had 80% power to detect the risk of MS with an odds ratio (*OR*) ≤ 0.77 corresponding to a one SD increase in fracture. Meanwhile, our MR study had 97% power to identify the risk of MS with β = −0.375 (*OR* = 0.69) corresponding to a one SD increase in fracture, as identified using the IVW method in Table 3.

### Multivariable MR analyses

Regarding the significant causal effect of fracture on MS, multivariable MR analyses showed that the causal effect of fracture on MS was independent of smoking (β = −0.311, *p* = 0.009), drinking (β = −0.383, *p* =0.003), and obesity (β = −0.371, *p* = 0.005), but dependent of BMD (β = −0.327, *p* = 0.245), as provided in Table 5.

**Table 5:** Multivariable MR analyses of the causal association of fracture on MS.

**Effects**	**Methods**	**Beta**	**95% CI (lower)**	**95% CI (upper)**	***P-*value**
Fracture on MS adjust for smoking	Multivariable IVW	−0.311	−0.544	−0.077	0.009
Fracture on MS adjust for drinking	Multivariable IVW	−0.383	−0.637	−0.13	0.003
Fracture on MS adjust for obesity	Multivariable IVW	−0.371	−0.633	−0.109	0.005
Fracture on MS adjust for BMD	Multivariable IVW	−0.327	−0.878	0.224	0.245

IVW, inverse-variance weighted; the thresholds for the statistically significant association and suggestive association are *p* < 0.05/4 = 0.0125 and *p* < 0.05, respectively.

## Discussion

Here, we performed a MR study to clarify the causal association between BMD/fracture and the risk of MS using the large-scale publicly available GWAS datasets from BMD, fracture, and MS (10, 11). We first evaluated the bidirectional causal effects of BMD and MS. The main analysis method IVW showed no significant causal effect of BMD on the risk of MS (β= 0.058, and *p* = 1.98E-01), and MS on the risk of BMD (β = −0.001, and *p* = 7.83E-01). We then evaluated the bidirectional causal effects of fracture and MS. However, we only identified a significant causal effect of fracture on the risk of MS using IVW (β = −0.375, *p* = 0.002), but no significant causal effect of MS on the risk of fracture using IVW (β = 0.011, *p* = 2.39E-01). Therefore, our main analysis method IVW only found a significant causal effect of fracture on MS using the threshold for the statistically significant association *p* < 0.05/4 = 0.0125. Meanwhile, multivariable MR analyses showed that the causal effect of fracture on MS was independent of smoking, drinking, and obesity, but dependent of BMD.

Bone metabolism in patients with MS is very complex (39). Different kinds of factors, such as physical activity, depression, and fatigue contributed to reduced femoral neck BMD in patients with MS independently (39). The supplementation with propionic acid may protect against osteoporosis in patients with MS (40). Growing evidence from observational studies showed the association between BMD/fracture and the risk of MS (2–8). A retrospective cohort study in 1,232 patients with MS and 12,320 matched controls showed more common primary hip fragility fractures in the MS cohort compared with the matched cohort (41). A recent systematic review and meta-analysis of 35 studies identified the pooled prevalence of osteoporosis to be 17% in 13,906 patients with MS (42). Another comprehensive systematic review and meta-analysis of 86 studies in the world including 103,334,579 people suggested the prevalence of osteoporosis to be 18.3% (43). Therefore, observational studies have identified inconsistent findings about the prevalence of osteoporosis or fracture in patients with MS and controls or the general population.

Our MR study shows that genetically increased BMD may increase the risk of MS using the contamination mixture method, but not the main analysis method IVW. It is noted that 307 BMD genetic variants were identified by large-scale GWAS in 142,487 individuals from the general population, UK Biobank (10). Therefore, our MR findings just reflect the effects of BMD on the risk of MS in the general population, may be not applicable to patients with low BMD, osteoporosis, or a history of fractures, as described in recent MR studies evaluating the serum calcium levels, BMD, and fractures (16, 44, 45). Meanwhile, the genetically increased BMD just represents lifelong exposure and may not directly equate with an intervention (46).

Compared with previous observational studies, our current study may have multiple strengths. First, we selected the large-scale BMD (142,487 individuals), fracture (185,057 cases and 377,201 controls), and MS (14,802 MS cases and 26,703 controls) GWAS datasets, as well as more genetic variants (307 for BMD, 15 for fracture, and 200 for MS) as the potential instrumental variables. These large-scale sample sizes and the number of genetic variants may provide enough statistical power. Second, we selected multiple MR analysis methods, such as the IVW, weighted median, MR-Egger, contamination mixture, and MR-PRESSO methods, which have been widely used in recent MR studies (21, 47–49).

In addition to these above strengths, our current study still has several limitations. First, we selected 307 heel BMD genetic variants as the potential instrumental variables. However, only 222 heel BMD genetic variants were available in the MS GWAS summary dataset. Second, we selected 200 MS genetic variants as the potential instrumental variables, but only 139 genetic variants were available in the fracture GWAS summary dataset. Third, we could not fully exclude the potential effects of pleiotropy. In fact, low BMD or fracture has many clinical risk factors, such as earlier menopause, rheumatoid arthritis, type I diabetes, inflammatory bowel disease, decreased thyroid stimulating hormone, increased homocysteine levels, decreased grip strength, late puberty, increased fasting glucose levels, coronary heart disease, type II diabetes, decreased vitamin D levels, and decreased dairy calcium intake (11). Meanwhile, there may be some common risk factors, such as smoking, drinking, obesity, and other unknown risk factors. Using three pleiotropy analysis methods, such as the MR-Egger intercept test, Cochran's *Q* statistic heterogeneity test, and the MR-PRESSO Global test, we have found evidence of pleiotropy when we evaluated the causal effects of BMD on MS, MS on BMD, and MS on fracture, as provided in Table 4. However, we did not find any pleiotropy when we evaluated the causal effects of fracture on MS, as provided in Table 4.

In summary, our MR analysis demonstrates that genetically increased fracture may reduce the risk of MS. Our findings should be verified and the underlying mechanisms should also be further evaluated by future studies.

## Data availability statement

The original contributions presented in the study are included in the article/Supplementary material, further inquiries can be directed to the corresponding author/s.

## Author contributions

YY and XW contributed to conception and design of the study. YY performed the statistical analysis and wrote the first draft of the manuscript. All authors contributed to manuscript revision, read, and approved the submitted version.

## Funding

This work was supported by funding from the Natural Science Foundation of Heilongjiang Province of China (JQ2020H003).

## Conflict of interest

The authors declare that the research was conducted in the absence of any commercial or financial relationships that could be construed as a potential conflict of interest.

## Publisher's note

All claims expressed in this article are solely those of the authors and do not necessarily represent those of their affiliated organizations, or those of the publisher, the editors and the reviewers. Any product that may be evaluated in this article, or claim that may be made by its manufacturer, is not guaranteed or endorsed by the publisher.
